# Impact of Different Irrigant Agitation Methods on Bacterial Elimination from Infected Root Canals

**DOI:** 10.3390/dj7030064

**Published:** 2019-06-27

**Authors:** Wajih Hage, Roeland J. G. De Moor, Désirée Hajj, Germain Sfeir, Dolla K. Sarkis, Carla Zogheib

**Affiliations:** 1 Department of Endodontics, School of Dentistry, Saint Joseph University, Beirut BP 17-5208, Lebanon; 2 Department of Conservative Dentistry and Periodontology, School of Dentistry, Medical University of Vienna, Vienna 1090, Austria; 3 Austrian Cluster for Tissue Regeneration, Vienna 1090, Austria; 4 Department of Endodontology, Dental School, Ghent University, Ghent B9000, Belgium; 5 Microbiology Laboratory, School of Pharmacy, Saint Joseph University, Beirut BP 17-5208, Lebanon; 6 Head, Department of Endodontics, School of Dentistry, Saint Joseph University, Beirut BP 17-5208, Lebanon

**Keywords:** biofilm, laser-activated irrigation, root canal irrigation, sonic activation, ultrasonic activation

## Abstract

Activation techniques are essential for root canal disinfection but may result in incomplete removal of bacteria. The aim of our study was to assess the antibacterial action of sonically, ultrasonically and laser-activated irrigation and 5.25% sodium hypochlorite (NaOCl) on *Enterococcus faecalis* in an infected tooth. Forty-four extracted mandibular premolars were mechanically prepared, sterilized, and inoculated with *E. faecalis* for 1 week. Bacterial counts after inoculation were evaluated in 4 randomly chosen teeth, remaining root canals were divided into 4 groups. Group A: laser-activated irrigation by photon-induced photoacoustic streaming, Group B: the sonic irrigation by EDDY, Group C: ultrasonic irrigation by EndoUltra, and Group D: 5.25% NaOCl. Colony forming unit (CFU) counts were measured and Kolmogorov–Smirnov, Wilcoxon, Kruskal–Wallis and Mann–Whitney tests were used to determine differences. The mean of CFU was found to significantly decrease in group D, 2110 ± 1015.93 (*p* < 0.001). Changes in measurement levels followed the same trend over time in groups A 27.40 ± 30.15, B 81.3 ± 85.68 and C 44.40 ± 67.12 (*p* = 0.141). The average CFU after irrigation in all groups was significantly greater than 0. Within the limitations of this study, all activation techniques were superior to NaOCl 5.25% in reducing *E. faecalis* from the infected tooth model.

## 1. Introduction

The aim of root canal therapy is to reduce, as much as possible, the bacterial population colonizing the root canal system [1]. Many species are associated with pulpal and peri-radicular pathology and some are even in direct correlation with endodontic failure [2]. Persistent bacteria in root canal systems are found to be the major source of failure [2]. 

*E. faecalis* is commonly detected in asymptomatic persistent endodontic infections as it possesses various survival and virulence factors [3]. In a recent systematic review, a higher correlation was found with persistent intraradicular infections compared with untreated chronic apical periodontitis [4]. In this respect a continued research on *E. faecalis* elimination from the root canal system is advocated [5].

Basic chemo-mechanical preparation with different sodium hypochlorite (NaOCl) concentrations leaves at least 40 to 60% of the initial bacterial counts in infected root canals and it is impossible to render root canals predictably free of cultivable bacteria [5,6,7]. The additional use of EDTA as a smear layer remover as well as an interappointment dressing with calcium hydroxide might increase the cases showing negative cultures [8]. This highlights the need for more effective strategies and the need to maximize the efficacy of irrigants that tend to induce better disinfection before filling the root canal systems [9]. In this regard, activation of irrigation techniques and delivery systems were introduced with the promise of optimizing the disinfection of root canals. An example of more recently introduced new activation techniques are: sonic activation (EDDY, VDW, Munich, Germany), ultrasonic activation (EndoUltra, MicroMega, Besancon, France) and laser activated irrigation with super short 50 microsecond pulse durations (PIPS, Fotona, Ljubljana, Slovenia).

EDDY (VDW, Munich, Germany) induces sonic oscillations using tips that are powered at a frequency of 6000 Hz by air scaler. The vibration produced is transferred to a polyamide tip that apparently does not damage the root canal walls [10].

EndoUltra (MicroMega, Besancon, France) is a hand-piece designed for intra-canal disinfection. It relies on ultrasonic technology with a frequency of 40 KHz, inducing acoustic streaming and cavitation. Its use aims to reduce bacterial levels through sonochemistry [11].

PIPS (Fotona, Ljubljana, Slovenia) is an acronym for photon-induced photoacoustic streaming. It can be described as an advanced laser activated irrigation (LAI) process using an Erbium laser (2940 nm) to pulse extremely low energy levels of laser light with short microsecond pulse duration to generate a photo-acoustic shock wave and where the tip is not positioned in the root canal itself [12]. It can stream irrigants throughout the entire root canal system [13,14]. It allows for better physical removal of an endodontic biofilm [15] as well as of a biofilm-mimicking hydrogel in an isthmus [16] when compared to ultrasonically activated irrigation.

To our knowledge, there is no study in the literature comparing the antibacterial effect of EDDY sonic, ENDOULTRA ultrasonic irrigation system with the PIPS laser activation tip_2_. Therefore, the aim of the present study was to evaluate the effectiveness of these recently introduced devices as compared to conventional irrigation in the reduction of *E. faecalis* populations.

## 2. Materials and Methods

The study protocol was approved by the “Ethics Committee” of Saint Joseph University Beirut (FMD158, 20-4-2018)

### 2.1. Specimen Selection and Preparation

Forty-four (4 groups of 10 teeth + 4 negative controls) freshly extracted single rooted mandibular premolars were selected for this study. The premolar crowns were intact with no previous restoration. The presence of a single root canal was determined by preliminary radiographs taken in both mesio-distal and bucco-lingual directions. All premolars demonstrating fractured or immature apices were excluded from this study.

The included premolars were scaled on the external root surfaces. They were placed in a saline solution of 0.9% at 4 °C for 24 h. Subsequently, samples were removed from the solution, and rinsed with distilled water.

After preparation of the opening cavity, the patency of each root canal was established using a 10 K-flexofile (Dentsply, Maillefer, Ballaigues, Switzerland) until it was visible through the apical foramen. Working length was established at 1 mm shorter than the apex. With this information in mind, the roots were further reduced in length, allowing a working length of 16 mm. Root canals were enlarged as per the manufacturer’s recommendation, sequentially up to a size of F2 Protaper Gold (Dentsply^®^, Maillefer, Switzerland). Alternatively, irrigation was performed with 5.25% NaOCl (Clorox, Vernon, CA, USA) during the whole shaping process with a 3 mL syringe and a 27G lateral side ject needle (Transcodent GmbH &Co., Sulzer, Switzerland) (0.05 mL/sec). A volume of 3 mL distilled water was used after the last NaOCl irrigation in order to remove the remaining solution. Then, a last flush of EDTA 17% (Vista, WI, USA) was used as a final irrigation (3 mL, 0.05 mL/sec for 1 min). Each root canal was dried with sterile paper points.

The foramen was sealed with resin-epoxy material in order to prevent bacterial leakage and teeth were mounted in silicone impression material blocks (3M ESPE Express STD, MN, USA).

The specimens were then sterilized for 20 min at 121 °C at 20 psi pressure (W&H-Lisa, Bürmoos, Austria).

### 2.2. Specimen Contamination

*E. faecalis* derived from ATCC 29212 was obtained from the microbiologic department of Saint Joseph university and cultured aerobically on blood agar at 35 °C for 48 h. Colonies were then grown in Brain Heart Infusion (BHI) broth at 37 °C for 24 h. Inoculum was prepared in sterile BHI broth and turbidity was set to 0.5 McFarland corresponding to approximately 1.5 × 10^8^ colony forming units per milliliter (CFU/mL). A total of 10 µl of the culture was immediately inoculated in the root canals. Teeth were placed in sterile cups and incubated at 37 °C for 7 days. Four root canals were randomly selected for enumeration of *E. faecalis* directly after inoculation. Inoculum was renewed every day following incubation to ensure maintenance of the culture viability.

### 2.3. Protocols for Irrigant Activation

After the incubation period, canal roots were divided into 4 groups:

Group A (n = 10)—laser-activated irrigation with photon-induced photoacoustic streaming (PIPS^®^): a 2.940 nm Er:YAG laser (Light Walker DT, Fotona, Ljubljana, Slovenia) equipped with a H14 handpiece (LightWalker Handpiece, Fotona, Ljubljana, Slovenia) holding a conical PIPS tip (9 mm long; 600 µm diameter) was used to activate the irrigant. The fiber tip was positioned at the entrance of the canal. The pulse energy 0.02 J, the frequency was 15 Hz, and the pulse duration was 50 microseconds. The irrigation protocol was as follows: the canal was flushed with 3 mL irrigant (0.05 mL/sec) using a 27G lateral side ject Needle (Transcodent GmbH and Co. KG, Kiel, Germany) while activating. The procedure was repeated 3 times. A total of 3 mL of distilled water followed the irrigation protocol.

Group B (n = 10)—sonically activated irrigation with EDDY (VDW, Munich, Germany): the tip made of flexible polyamide with a size of 25.04, driven by an air hand piece (AirScaler-W&H, Lisa, Bürmoos, Austria) was moved up and down over a distance of 3 mm starting 1 mm from the apical terminus without pressure according to the manufacturer’s recommendations (6000 Hz, 0.3 mPa/3 bar) for 30 s. The procedure was repeated 3 times. In between each activation cycle, the canal was flushed with 3 mL irrigant (0.05 mL/sec) (NaOCl 5.25%) using a 27G needle.

Group C (n = 10)—ultrasonic irrigation with EndoUltra (MicroMega, Besancon, France): this cordless device oscillates an activator tip of 15/0.2 at 40 KHz, the system was performed using 5 mL of 5.25% NaOCl solution in a vertical movement over a distance of 3 mm without pressure for 30 s starting 1 mm from the apical terminus according to the instructions of the manufacturer. The procedure was repeated 3 times. In between each activation cycle, the canal was flushed with 3 mL irrigant (NaOCl 5.25%) using a 27G needle.

Group D (n = 10)—conventional irrigation: a 27G needle with a lateral exit was applied without pressure in a vertical movement of 3 mm, at 1 mm from the apical end of the preparation, using a 27G lateral side ject needle (Transcodent GmbH &Co. KG, Kiel, Germany) of 3 mL of NaOCl 5.25% for 5 min (0.05 mL/sec). This constituted the control group.

Following irrigation, after drying the root canals, *E. faecalis* bacterial count was evaluated by placing a sterile paper point into each canal for 5 min. Paper points were then placed in 500 µL sterile BHI broth for 15 min. After mixing by vortex, 50 µL of the liquid medium was serially diluted in sterile BHI broth and plated on blood agar. Culture media was placed at 37 °C for 48 h. Colonies were counted and confirmed by colony morphology observation on blood agar and Gram staining.

Non-inoculated root canals were similarly incubated and cultured to serve as negative control (n = 4).

### 2.4. Statistical Analysis

The Statistical Package Software for Social Science (SPSS for Windows, Version 22.0, Chicago, IL, USA) was used to perform the statistical analysis. The level of significance was set at *p* ≤ 0.05. Kolmogorov–Smirnov tests were conducted to evaluate the normality distribution of continuous variables. Wilcoxon tests were performed to compare CFU before and after irrigation for each group. The variation of CFU after irrigation was compared between the 4 groups using Kruskal–Wallis tests, followed by Mann–Whitney tests.

## 3. Results

**Figure 1:**
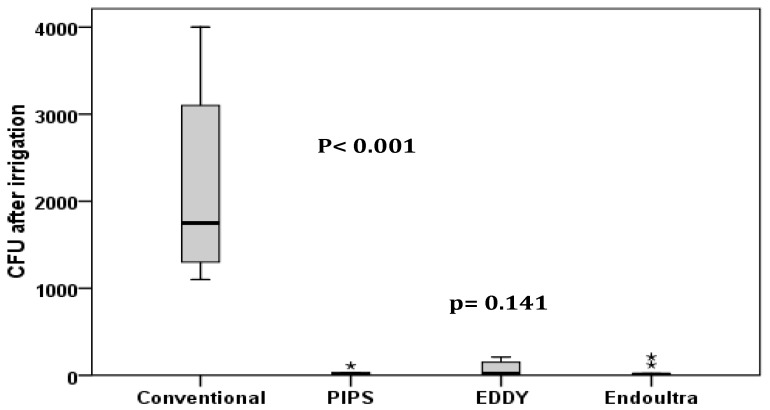
Distribution of CFU among groups. The decrease of CFU was significantly lower after conventional irrigation (*p* < 0.001). However, no significant difference was found between photon-induced photoacoustic streaming (PIPS), EDDY and EndoUltra (*p* = 0.141).

In Table 1, the mean and SD of the CFU in each group are shown. The data are also represented in box plots in Figure 1. The CFU has decreased significantly after canal irrigation with the conventional method (*p* < 0.001), PIPS (*p* < 0.001), EDDY (*p* < 0.001) and EndoUltra (*p* < 0.001).

The decrease of CFU was significantly lower after conventional irrigation (*p* < 0.001). The effect was more pronounced with the laser activated irrigation but changes in measurements between groups followed the same trend with no statistically differences between PIPS, EDDY and EndoUltra (*p* = 0.141).

The percentage of decrease was high but significantly different from 100% (*p* < 0.05), which means that no irrigation technique can completely eliminate bacterial colonies of *E. faecalis* from root canals.

## 4. Discussion

Disinfection of the root canal system is a necessity during the chemomechanical preparation. Multiple shaping instruments are available with satisfactory mechanical and physical properties, though not capable of cleaning entirely the root canal wall [17,18]. A chemical disinfection of the root canal system is mandatory for the removal of the majority of bacterial populations that are present in infected root canals. Next to the amount of uninstrumented root canal wall areas anatomical complexities found in the root canal system, especially in the apical third, that cannot be reached by instruments, require cleaning and disinfection with irrigation solutions [19]. A sodium hypochlorite solution (1–5.25%) used as root canal irrigant is considered the gold standard for root canal cleaning and disinfection [20].

It is very efficient in removing the infected pulpal tissues due to its proteolytic properties. It is known to be very effective against a large spectrum of bacteria, viruses, fungi and other microorganisms [21]. Moreover, some recent studies have shown that activating the sodium hypochlorite solution is also very effective in optimizing its capabilities [22].

The aim of our study was to assess the influence of three activation techniques in removing the bacterial population of *E. faecalis* from the root canal, compared to conventional needle syringe irrigation. The null hypothesis that the activated irrigation groups do not differ from each other in antimicrobial efficacy is not rejected.

This study was based on mandibular premolars with a single root canal. The samples were standardized in order to reduce the bias of the study. All the working lengths were set at 16 mm. The final apical taper was 8% and the final apical diameter for all the selected premolars was 0.25 mm.

In the present study, we tried to simulate the typical clinical scenario of the canal surinfection by renewing the inoculum daily for a week, as it is well known that when a tooth is infected, the pathology can easily amplify if the cause is not treated [23,24].

Root canal infection is typically caused by multiple microorganisms forming a well-organized biofilm [25]. The choice of *E. faecalis*, as a unique bacterium, was based on its behavior in infected root canals. It endures a prolonged period of nutritional deprivation. It binds to dentin and invades dentinal tubules. It alters the host response and suppresses the action of lymphocytes. *E. faecalis* possesses lytic enzymes, cytolisin, aggregation substances, pheromones and lipoteichoic acids [3,26]. Based on what preceded, *E. faecalis* can persist in the root canal system and peri-radicular lesions and is a common cause of failure of endodontically treated teeth [27].

The present set-up is one of the most common biofilm model systems [16]. In this respect, sampling after implementation of the investigated irrigation protocols was done with a sterile paper point. Loosening biofilm bacteria was not performed with a scraping action of a file along the root canal walls, though the paper point was left 5 min in the root canal [28].

Our results indicated that all activation techniques used in this study significantly lowered the CFU of *E. faecalis* compared to the initial ratio. Additionally, the conventional needle protocol is significantly less efficient in eliminating *E. faecalis*, when compared to the three irrigant agitation modalities. This result may be explained by the fact that conventional needle syringe irrigation provides far lower fluid dynamics as compared to the investigated activation techniques [14] and this also despite of the irrigation period of 5 min. There is also no increase of the NaOCl solution reaction rate which is seen with activation of irrigation solutions [29]. Furthermore, its use at 3 mm of the apical foramen may also induce a vapor-lock effect, therefore lowering the efficiency of NaOCl [30], as shown in a number of recent studies [31,32,33]. This was avoided by placing the needle at 3 mm from the apical end of the preparation length.

No statistically significant differences regarding the decrease of the CFU in the root canals activated by EDDY, EndoUltra and PIPS were found. This is consistent with a number of recent studies [34,35,36].

In general, it is believed that sonic activation is seemingly less effective than the use of ultrasound, as a more velocious fluid stream is induced with the latter [37,38,39,40]. Furthermore, instrument size, tip diameter, instrument taper, confinement of the instrument within the confines of the canal and the type of irrigant, all do have an influence on the cleaning and disinfection efficacy associated with enhanced fluid streaming. Although EDDY is categorized as a sonically activated system, the cleaning efficacy when evaluating the removal of a biofilm mimicking hydrogel (BMH) out of an isthmus, appeared to be at the same level as the PIPS approach [41]. In the same investigation, EDDY activation resulted in a higher removal of this BMH as compared to ultrasonic active irrigation (UAI) with the Irrisafe. Where sonic activation in general is described with a one node pattern, high speed imaging showed the EDDY moving in a circular direction and with the instrument having an additional extra movement of the most apical part of the tip, as is seen with the moving trunk of an elephant [42]. This specific agitation pattern also explained the higher cleaning efficacy than that of the EndoActivator in the latter investigations.

The literature regarding EndoUltra is limited. EndoUltra resulted in a more effective smear layer removal than the EndoActivator [43]. At this moment no further information is found on the EndoUltra. No studies on the antibacterial action are published to our knowledge.

The mechanical effect of LAI was shown to be stronger than ultrasonic activation during the physical removal of a biofilm-mimicking hydrogel (water as irrigant) [14] and of a dual species biofilm (*S. mutans* and *E. faecalis*) (saline as irrigant) [15], which was attributed to the extremely turbulent action of the irrigation solutions activated with a pulsed erbium laser; consequently, when NaOCl was used as an irrigant, the reductions were superior to the results obtained with saline and both LAI and ultrasound did no show significant differences any more [15]. 15Although not statistically significant, together with data from other studies the final number of bacteria left was lower, the final amount of reduction was higher or more negative samples were seen with LAI [31,34,36,44,45,46,47,48]. The same phenomenon is also seen in the present study. All irrigant activation techniques may be associated with apical irrigant extrusion causing post-operative undesirable outcomes. Data from previous in vivo investigation [49] indicated that all irrigation protocols gave satisfactory results in meaning of post-operative pain, without any significant difference noted with PIPS as a final irrigation technique.

In this study all three activation techniques resulted in similar antibacterial efficacy, demonstrating that the specific movement of the EDDY during irrigant activation brought the sonic approach at the same level as UAI with UltraEndo and LAI with the PIPS approach. Laser activation of 5.25% sodium hypochlorite significantly improved the cleaning of intracanal *E. Faecalis* followed by ultrasonic, and sonic activation with no statistical difference between the groups.

Further research is needed in order to evaluate the impact of specific fluid streaming patterns during irrigant activation on the interaction with endodontic biofilms.

## 5. Conclusions

Although none of the treatment regimens were able to reliably render canals sterile under the conditions used, laser activation of 5.25% sodium hypochlorite significantly improved the cleaning of intracanal *Enterococcus Faecalis* followed by ultrasonic, and sonic activation with no statistical difference between the groups. The impact of irrigant streaming related to different types of sonic and ultrasonic activation on biofilm interaction and removal needs further investigation.

## Figures and Tables

**Table 1:** Mean and Standard deviation of colony forming unit (CFU) in each group.

	Conventional	PIPS	EDDY	Endoultra	-*p*-Value
Control groups	1900000 ± 0.000	1700000 ± 0.000	1500000 ± 0.000	1500000 ± 0.000	1.000
After irrigation	2110 ± 1015.93 ^b^	27.40 ± 30.15 ^a^	81.30 ± 85.68 ^a^	44.40 ± 67.12 ^a^	<0.001
-*p*-value	<0.001	<0.001	<0.001	<0.001	
Percentage of decrease	99.889 % ± 0.053 ^a^	99.998% ± 0.002 ^b^	99.994% ± 0.006 ^b^	99.997% ± 0.004 ^b^	<0.001

(^a^, ^b^ Different letters indicate the presence of significant difference between irrigation groups).
